# Epstein-Barr virus gastric ulcer associated with ruxolitinib

**DOI:** 10.1007/s00277-016-2748-1

**Published:** 2016-07-14

**Authors:** Yoshiharu Kusano, Yasuhito Terui, Kyoko Ueda, Kiyohiko Hatake

**Affiliations:** Department of Hematology and Oncology, Cancer Institute Hospital, Japanese Foundation for Cancer Research, 3-8-31, Ariake, Koto-ward, Tokyo, Japan 135-8550

Dear Editor,

The Janus kinase 1 and 2 selective inhibitor ruxolitinib improves splenomegaly and overall survival of patients with primary myelofibrosis (PMF); however, opportunistic infections, although rare, can be a problem [1–4]. Here, we present the case of a patient who suffered from an Epstein-Barr virus (EBV) gastric ulcer due to severe immunosuppression induced by ruxolitinib. We treated the patient with ganciclovir, which proved completely effective against the EBV gastric ulcer.

**Fig. 1:**
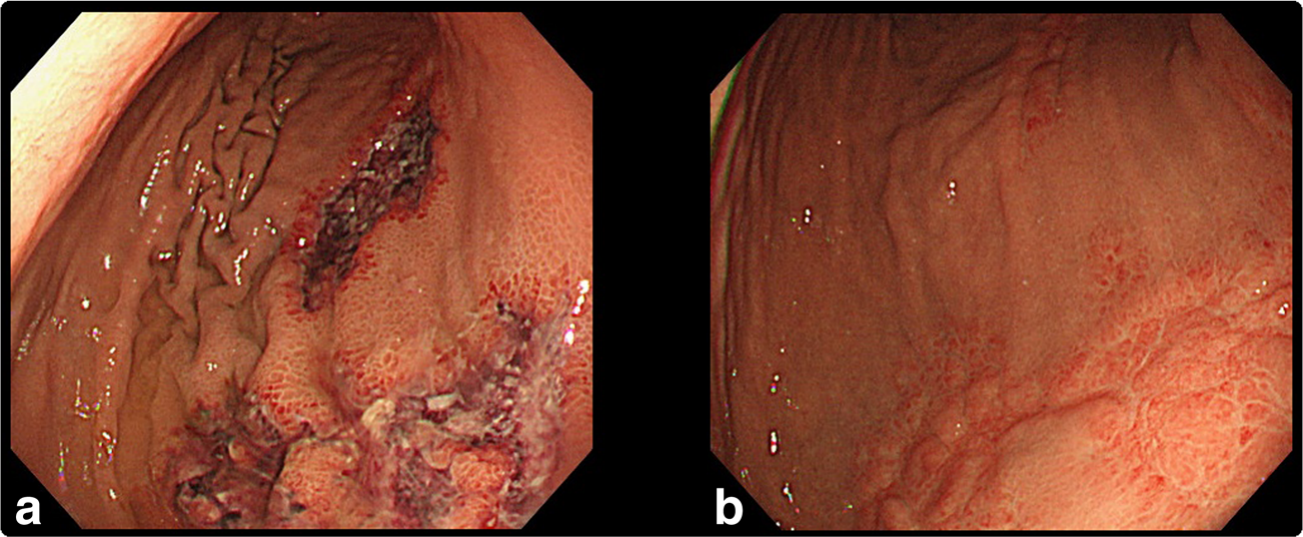
Endoscopic appearance of EBV gastric ulcer. **a** Endoscopic appearance before the treatment. **b** Endoscopic appearance after the treatment

A 77-year-old man with a 2-year history of PMF presented with severe diarrhea. Six months earlier, ruxolitinib had been initiated at 20 mg twice daily. PMF-associated manifestations had improved rapidly, but it had been necessary to taper the amount of ruxolitinib step by step to 5 mg twice daily because of repeated infections, including that of herpes zoster. The number of T cells and activity of natural killer (NK) cells had been decreasing. When his wife brought him to the hospital, he felt only mild discomfort in the epigastric area without tenderness by palpation despite experiencing numerous episodes of diarrhea (approximately 20 times per day). A computed tomography scan showed massive thickening of the gastric wall. An upper gastrointestinal endoscopy revealed multiple open-type gastric ulcers (Fig. 1a). No cytomegalovirus, herpes simplex virus, or EBV was detected in the plasma, but severe impairment of cell-mediated immunity was observed: CD4+ T cells at 123/μL, CD8+ T cells at 159/μL, and NK cell activity of 7 %. He both HIV-1 and HIV-2 antibodies were negative. Although a stomach specimen showed no indicative features, a viral infection was strongly suspected. Ruxolitinib was discontinued, and empiric ganciclovir administration was initiated. The following day, the frequency of diarrhea diminished to two or three episodes per day. EBV DNA was amplified with polymerase chain reaction from tissue of the gastric ulcer. Fourteen days of ganciclovir improved the patient’s general appearance, and he was discharged.

To date, evidence supporting the use of ganciclovir against EBV infections has been limited [5]. However, the gastric ulcer was completely cured with a further 7-day treatment with valganciclovir (Fig. 1b). However, although ruxolitinib was discontinued, the level of T cells and NK cell activity continued to decline even after 1 month: CD4+ T cells to 78/μL, CD8+ T cells to 75/μL, and NK cell activity to 1 %. Three months after the discontinuation of ruxolitinib, cell-mediated immunity began to slowly recover but remained low; in particular, NK cell activity remained at 1 %.
